# The Role of RIPC in Preventing Organ Damage, Inflammation, and Oxidative Stress during Lower Limb DSA: A Randomised Controlled Trial

**DOI:** 10.1155/2021/6043550

**Published:** 2021-12-08

**Authors:** Karl Kuusik, Teele Kasepalu, Mihkel Zilmer, Jaan Eha, Mare Vähi, Liisi Anette Torop, Jüri Lieberg, Jaak Kals

**Affiliations:** ^1^Department of Cardiology, Institute of Clinical Medicine, University of Tartu, Puusepa 8, Tartu 50406, Estonia; ^2^Heart Clinic, Tartu University Hospital, Puusepa 8, Tartu 50406, Estonia; ^3^Department of Biochemistry, Institute of Biomedicine and Translational Medicine, Centre of Excellence for Genomics and Translational Medicine, University of Tartu, Puusepa 8, Tartu 50406, Estonia; ^4^Institute of Mathematics and Statistics, University of Tartu, J. Liivi 2, Tartu 50409, Estonia; ^5^Pathology Service, Tartu University Hospital, Puusepa 8, Tartu 50406, Estonia; ^6^Department of Vascular Surgery, Surgery Clinic, Tartu University Hospital, Puusepa 8, Tartu 50406, Estonia; ^7^Department of Surgery, Institute of Clinical Medicine, University of Tartu, Puusepa 8, Tartu 50406, Estonia

## Abstract

**Objective:**

Diagnostic digital subtraction angiography (DSA) and DSA with percutaneous transluminal angioplasty (DSA-PTA) are common procedures for diagnosing and treating symptomatic lower extremity arterial disease (LEAD). However, organ damage following DSA and DSA-PTA is often underrecognised and hence undiagnosed. To reduce the risk induced by invasive procedures in symptomatic LEAD patients, the method of remote ischemic preconditioning (RIPC) has been suggested. The aim of the current study was to assess the effect of RIPC intervention on the organ damage markers profile, oxidative stress, and inflammation biomarkers in LEAD patients undergoing DSA and DSA-PTA procedure.

**Methods:**

The RIPC intervention was performed by inflating a standard blood pressure cuff on the patient's upper arm to 200 mmHg for 5 minutes four times with 5-minute perfusion between each cycle. The sham intervention was performed similarly, but the cuff was inflated to 20 mmHg. Changes in the cardiac and renal damage biomarkers' profile, oxidative stress, and inflammation biomarkers were recorded before and 24 hours after DSA or DSA-PTA.

**Results:**

A total of 111 (RIPC 54, sham 57) patients with symptomatic LEAD scheduled for endovascular procedure were randomised, and 102 patients (RIPC 47, sham 55) completed the study protocol. RIPC significantly limited the increase of adiponectine levels after DSA and DSA-PTA, compared to sham intervention (*p* = 0.020), but CK-MB levels were markedly lower in the sham group (*p* = 0.047) after procedure. There was no significant difference between the RIPC and the sham group in mean changes in hs-troponin-T (*p* = 0.25), NT-proBNP (*p* = 0.24), creatinine (*p* = 0.76), eGFR (*p* = 0.61), urea (*p* = 0.95), beta-2-microglobuline (*p* = 0.34), or cystatine C (*p* = 0.24) levels.

**Conclusion:**

In this controlled clinical study, RIPC failed to improve the profile of renal and cardiac biomarkers in patients with LEAD periprocedurally. RIPC significantly limits the rise in adiponectin levels and may influence the decrease of CK-MB levels 24 hours after endovascular procedure.

## 1. Introduction

Patients with symptomatic lower extremity artery disease (LEAD) often have generalized atherosclerotic disease with heart and kidney involvement and a significantly greater risk for cardiovascular death [1]. Digital subtraction angiography (DSA) is one of the possible techniques performed in symptomatic LEAD patients to determine the exact site of atherosclerotic lesion and its width. It is often followed up by endovascular treatment, such as percutaneous transluminal balloon angioplasty and/or stenting (PTA). Endovascular therapy is usually preferred as it is considered to be safer and better tolerated than open surgery. However, DSA and DSA-PTA have several drawbacks. It has been shown that balloon angioplasty and stent placement provoke inflammatory response in LEAD patients [2]. Moreover, contrast media administration during angiographic procedure reduces perfusion and induces hypoxia in renal medulla [3]. This contributes to contrast media's direct nephrotoxic effect and may lead to acute kidney injury [3]. To lower the risk of organ damage induced by treatment in patients with LEAD, additional therapies should be considered.

Remote ischaemic preconditioning (RIPC), a method of performing short repeated ischaemic episodes in a distant organ such as an upper limb, has been proposed to reduce organ damage induced by ischaemia-reperfusion injury (IRI). The exact mechanism of how RIPC reduces IRI and protects against organ damage is complex, partly still under debate, and remains beyond the scope of this study. Improvement in blood flow, avoiding mitochondrial dysfunction, and modifying gene expression and inflammatory response have been described among other changes [4, 5]. RIPC has been shown to be a safe method for inducing additional organ protection under prolonged ischaemic conditions in clinical situations periprocedurally, e.g., in patients who undergo angiographic procedures or vascular surgery. We have previously shown that RIPC may modulate arterial stiffness parameters and improve vascular function in patients after endovascular procedure [6]. We have also shown that RIPC reduces renal and cardiac damage inflicted by open vascular surgery [7, 8]. Thus, we hypothesised that RIPC may also reduce organ damage and inflammatory biomarkers in LEAD patients undergoing angiography. The aim of the current study was to assess the effect of RIPC intervention on the organ damage markers' profile, oxidative stress, and inflammation biomarkers in LEAD patients undergoing endovascular procedure.

## 2. Methods

### 2.1. Trial Design

Hospitalised patients scheduled for lower limb DSA or DSA-PTA with diagnosed LEAD were included in a double-blinded single-centre randomised controlled trial in a nonconsecutive manner. The primary outcome, reported in [6], was to compare the effect of RIPC and the effect of sham procedure on arterial stiffness and on the haemodynamic profile. The secondary outcome, based on the present study, was to assess the effect of RIPC on cardiac and renal injury, oxidative stress, and inflammation biomarkers in patients with LEAD. The trial was approved by the Research Ethics Committee of the University of Tartu and was registered at the U.S. National Institute of Health and the U.S. National Library of Medicine clinical trials register (ClinicalTrials.gov) (identifier: NCT02700958).

### 2.2. Participants

Patients with symptomatic LEAD scheduled for DSA or DSA-PTA provided written informed consent in their native language. All patients were recruited from the Department of Vascular Surgery, Tartu University Hospital, Estonia, between February 2016 and March 2018. The exclusion criteria were age under 18, estimated glomerular filtration rate measured at admission to hospital less than 30 ml/min/1.73 m^2^, simultaneous participation in any other clinical trial, coexisting pathology of the upper limbs limiting the use of the cuff, active malignant tumour (in remission for less than 5 years or ongoing treatment), documented allergic reaction to iodinated contrast agent, acute infection (body temperature 38 degrees of Celsius or higher, C-reactive protein 50 mg/L or higher), cardiac rhythm abnormalities (atrial fibrillation, frequent supraventricular, and ventricular complexes), home-based oxygen treatment, inability to lie supine for 40 minutes, vascular surgery in the axillary region, and documented upper limb deep vein thrombosis.

### 2.3. Randomisation

Randomisation was carried out by applying the stratified permuted-block randomisation technique. Six strata were formed combining age in years (≥75 or <75) and estimated glomerular filtration rate (≥90, 60-89, or 30-59, ml/min/1.73 m^2^). Block size was set to randomly permutate between 2 and 4. Randomisation sequence was conducted prior to the beginning of the study, using the WINPEPI computer program, and the labels were manually sealed into nontransparent envelopes tagged by the number of strata and sequentially numbered order by the study group's randomiser. The envelope based on the data of the participant's age and estimated GFR on admission to hospital was opened immediately before the initiation of the intervention by the study director.

### 2.4. Interventions

The RIPC intervention was performed by the study director by inflating a standard blood pressure cuff on the patient's upper arm to 200 mmHg for 5 minutes four times with 5-minute perfusion between each cycle. If the patient's systolic blood pressure was higher than 180 mmHg before intervention, the blood pressure cuff was filled to 20 mmHg above systolic pressure. The sham intervention was accomplished by inflating the cuff to 20 mmHg for 5 minutes in 4 cycles and 5-minute perfusion time between each cycle. Interventions were applied as close as possible to the subsequent endovascular procedure.

### 2.5. Blinding

The participants were blinded to the applied intervention by concealing the cuff's pressure gauge. The medical personnel in charge of the treatment of the participants were informed about their patients' consent to participate in the trial, but were kept blinded to the applied intervention. Measurements of biomarker levels from blood and urine samples were made without the knowledge of the assigned intervention. Statistical models were developed, and analysis was made without the knowledge of the assigned intervention.

### 2.6. Outcomes

Blood samples were collected in the morning before the intervention and 24 hours after procedure. Urine samples were collected twice: in the morning before the intervention and 24 hours after DSA or DSA-PTA. Blood and urine samples were collected in the fasted state, at least 3 hours since the last meal. Cardiac biomarkers (NT-proBNP, hs-CRP, CK-MB, and troponin T), renal biomarkers (urea, creatinine, cystatin C, beta-2-microglobulin, eGFR, and NGAL), and biomarkers of oxidative stress and inflammation (hs-CRP, IL-18, ox-LDL, adiponectine, and myeloperoxidase) were measured from all blood samples. Urinary renal biomarkers (L-FABP, KIM-1) and biomarker of oxidative stress and inflammation (isoprostanes over urine creatinine ratio) were measured from all urine samples.

### 2.7. Sample Size

Sample size was calculated based on the primary outcome of this trial, augmentation index corrected to heart rate of 75 beats per minute, where 47 patients in both intervention groups were considered adequate. No power analysis was performed for organ damage markers.

### 2.8. Statistical Methods

Intention-to-treat (ITT) analysis was performed for all participants who received the allocated intervention. The arithmetic mean imputation method for missing outcomes was used to complete the data for ITT analysis. Subsequently, per-protocol analysis (PP) was conducted to support ITT analysis. In addition, individual markers were adjusted to the corresponding baseline values. As ITT is known to take into account both known and unknown factors, no adjusting to baseline was considered necessary in ITT analysis.

Continuous variables were compared using Student's *t*-test or the Wilcoxon rank-sum test as appropriate. For statistical analysis of multiple repeated measures, one-sided or two-sided analysis of variance (ANOVA) was used where appropriate. Categorical variables were compared with the Chi-squared test.

## 3. Results

**Figure 1:**
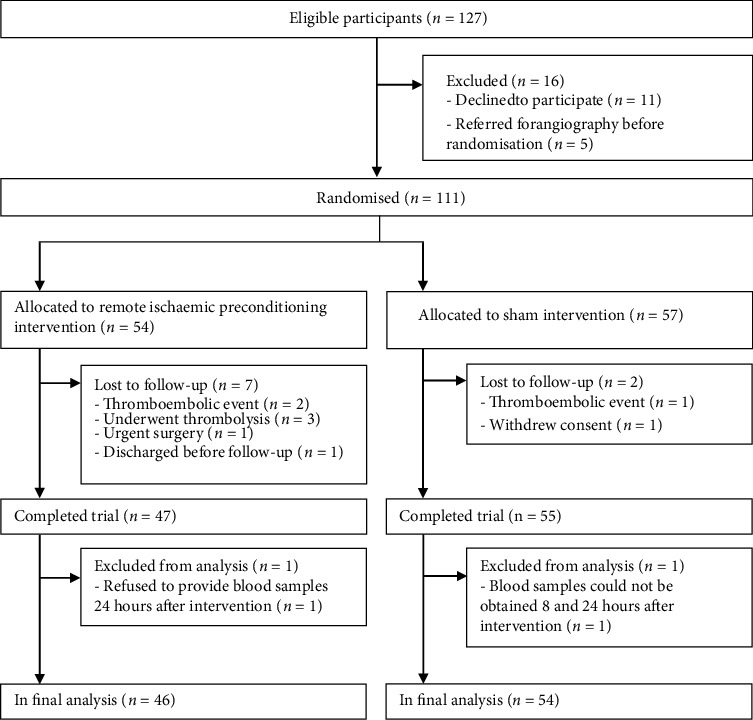
Flow diagram of patient enrolment.

Altogether, 127 eligible nonconsecutive patients were invited to participate in the trial. Fifty-four patients were allocated to the RIPC group and 57 to the sham group. Forty-seven patients of the RIPC intervention group and 55 of the sham group completed the trial (Figure 1). In total, 111 patients were included in ITT analysis, and 100 (90%) patients were included in PP analysis. The baseline values of pro-BNP and NGAL were significantly higher for the RIPC vs. sham group in the PP population, but not in the ITT population. No other significant difference was noted in baseline characteristics between the population of per protocol analysis (Table 1) and the population of ITT analysis (Table S1).

The median time from the beginning of intervention to the beginning of DSA or DSA-PTA was 80 minutes (IQR 60-118) in the RIPC group and 79 minutes (IQR 64-112) in the sham group (*p* = 0.377). There was no significant difference between the RIPC and the sham intervention regarding the time spent for endovascular procedure (*p* = 0.108) or the time from the beginning of intervention to the time blood was collected (23 h 49 min and 24 h 13 min; *p* = 0.178, respectively). Of the patients included in the final analysis, 21 (45.7%) patients from the RIPC group and 22 (40.7%) patients from the sham group received only DSA (*p* = 0.621), at least one stent was placed to 22 (47.8%) patients in the RIPC group and to 30 (55.6%) patients in the sham group (*p* = 0.44).

### 3.1. Changes in Oxidative Stress and Inflammation Markers

There was no significant change in hs-CRP levels between the RIPC group and the sham group in primary analysis (*p* = 0.45). Both in the RIPC (*p* < 0.0001) and the sham (*p* = 0.03) group, a significant increase in hs-CRP levels was noted 24 hours after intervention. After adjusting PP analysis to baseline values, a significant increase in hs-CRP levels occurred only in the RIPC group (*p* = 0.002), but not in the sham group (*p* = 0.40). hs-CRP levels were significantly higher in the patients of both the RIPC group (*p* = 0.036) and the sham group (*p* < 0.0001) who had received stents (Table 2).

In primary analysis, a significant increase in IL-18 levels occurred only in the sham group (*p* = 0.020), but not in the RIPC group (*p* = 0.88). There were no significant changes in IL-18 or ox-LDL levels when the RIPC group was compared to control.

**Figure 2:**
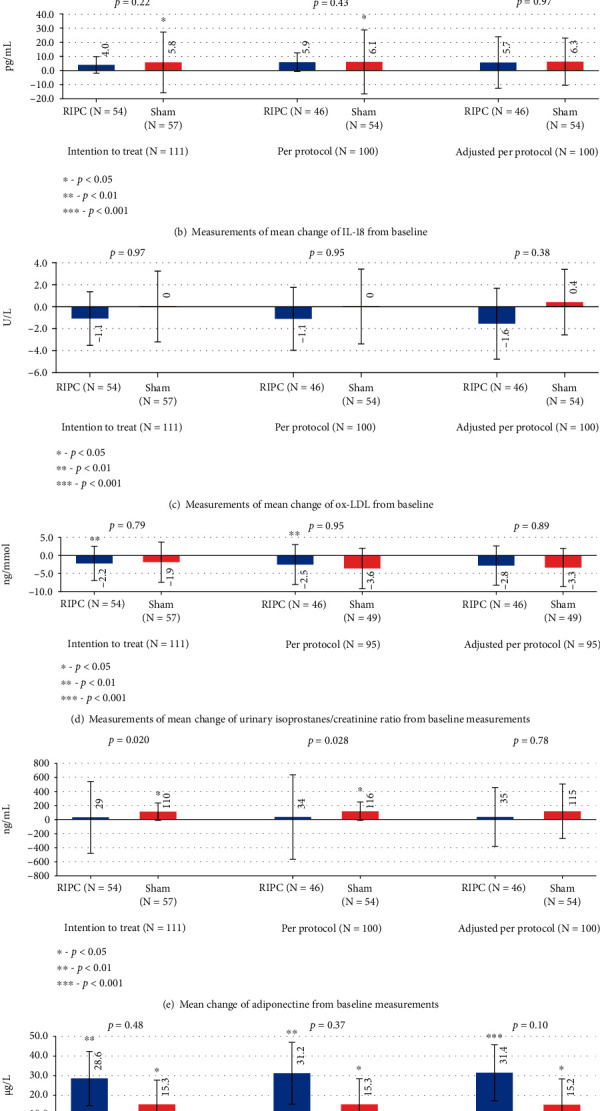
Mean changes of oxidative stress and inflammation biomarkers. Error bars represent the confidence interval for the mean.

We did not see a significant change in the levels of urinary isoprostanes corrected for creatinine in the RIPC vs. sham group (*p* = 0.786), but a significant decrease in the isoprostanes-creatinine ratio was noted only in the RIPC group both in ITT (*p* = 0.008) and in PP (*p* = 0.008) analysis (Figure 2).

Primary analysis revealed a significant increase in adiponectine levels only in the sham group (*p* = 0.04). A significant difference in adiponectine levels was seen between the RIPC group and the sham group both in primary (*p* = 0.020) and PP analysis (*p* = 0.028).

MPO levels were significantly increased in both groups 24 hours after intervention (*p* = 0.007 in the RIPC group; *p* = 0.015 in the sham group), but there was no significant difference between the groups (*p* = 0.015) in primary analysis. Similar changes occurred also in the PP population before and after adjusting to baseline values (Figure 2).

### 3.2. Changes in Cardiac Biomarkers

**Figure 3:**
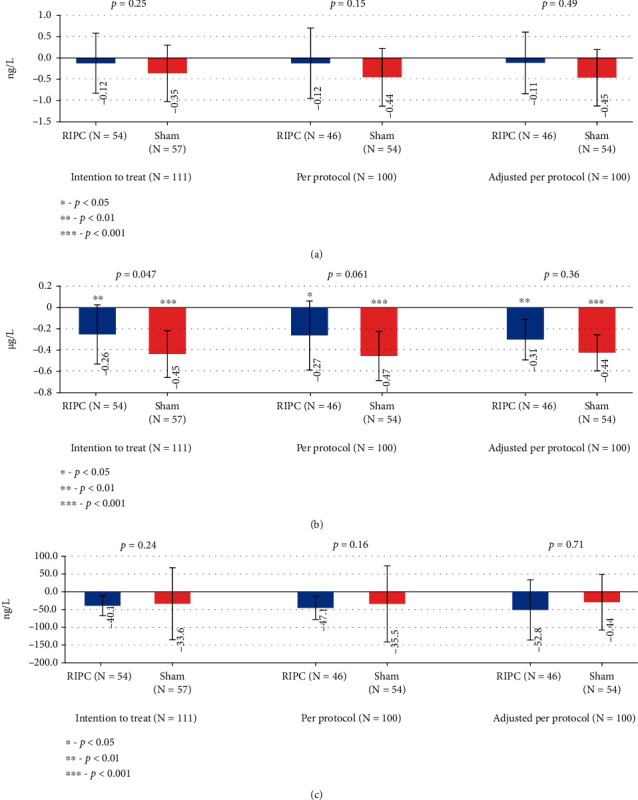
Mean changes of cardiac biomarkers. Error bars represent the confidence interval for the mean.

There was no significant difference between the RIPC and the sham group in mean changes of hs-Troponin-T (*p* = 0.25) and NT-proBNP (*p* = 0.24) levels (Figure 3). A significant decrease in CK-MB occurred both in the RIPC (-0.26 *μ*g/L; *p* = 0.009) and sham (-0.45 *μ*g/L; *p* < 0.0001) groups and was greater in the sham group (*p* = 0.047). This difference, however, was not found either before (*p* = 0.061) or after adjusting to baseline values (*p* = 0.36) in PP analysis (Figure 3).

### 3.3. Changes in Serum Kidney Function Markers

**Figure 4:**
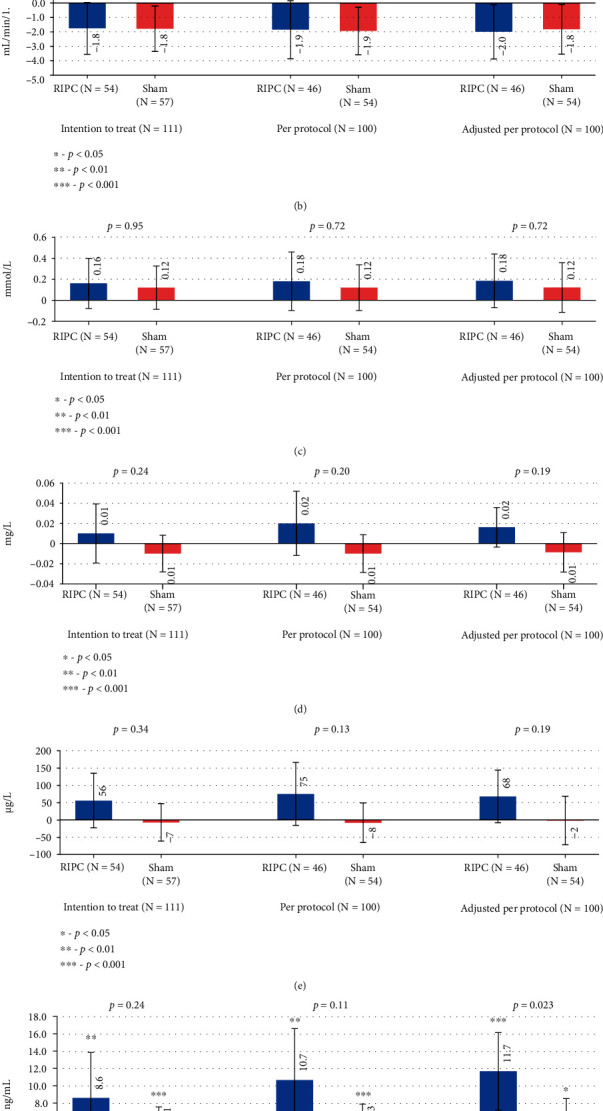
Mean changes of serum kidney biomarkers. Error bars represent the confidence interval for the mean.

The increase in creatinine levels was seen both in the RIPC (*p* = 0.050) and sham (*p* = 0.032) groups. RIPC did not significantly reduce the rise in creatinine (*p* = 0.76) levels compared to control (*p* = 0.76) (Figure 4). No significant change was noted in eGFR (*p* = 0.61), urea (*p* = 0.95), beta-2-microglobuline (*p* = 0.34), or cystatine C (*p* = 0.24) levels. A decrease in eGFR was revealed in the sham group in primary analysis (-1.79 mL/min/1.73 m^2^; *p* = 0.024). This finding was supported by PP analysis (-1.94; mL/min/1.73 m^2^; *p* = 0.015). After adjusting to baseline values, the decrease in eGFR levels from baseline was evident in both groups: -1.99 mL/min/1.73 m^2^ in the RIPC group (*p* = 0.040) and -1.82 mL/min/1.73 m^2^ in the sham group (*p* = 0.042) (Figure 4).

In primary analysis, a significant increase in NGAL levels was seen both in the RIPC (8.6 ng/ml; *p* = 0.002) and in the sham group (5.1 ng/ml; *p* = 0.002); however, this change was not significant between the groups (*p* = 0.24) and remained also insignificant in PP analysis (*p* = 0.11). After adjusting to baseline values, a significant increase in NGAL levels in the RIPC vs. sham group was noted for the PP population (*p* = 0.023) (Figure 4).

### 3.4. Changes in Urinary Kidney Injury Markers

**Figure 5:**
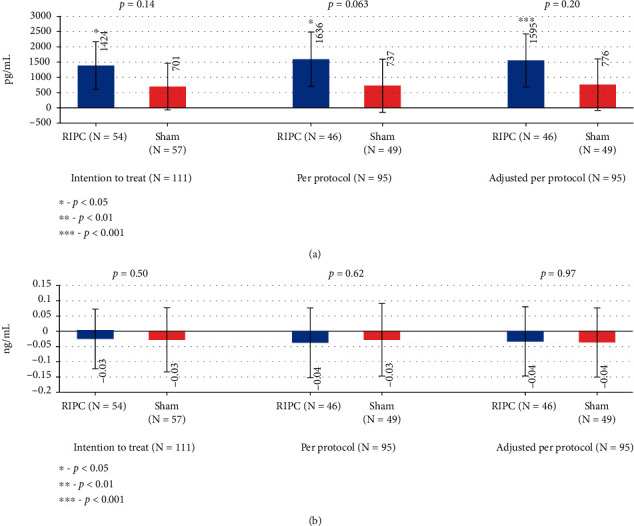
Mean change of urinary kidney biomarkers. Error bars represent the confidence interval for the mean.

A significant increase in KIM-1 levels was found in the RIPC group (*p* = 0.011), but not in the sham group (*p* = 0.092). Similar changes were also revealed in PP analysis before and after adjusting to baseline values. There were no significant differences in KIM-1 (*p* = 0.14) or L-FABP (*p* = 0.20) levels between the RIPC group and the sham group (*p* = 0.14) in primary analysis (Figure 5).

## 4. Discussion

In this controlled clinical study, RIPC failed to improve the profile of renal and cardiac biomarkers of patients with LEAD periprocedurally. On the other hand, we showed that RIPC significantly limits the increase in adiponectin levels and may affect the decrease in CK-MB levels 24 hours after endovascular procedure.

### 4.1. Effect of Contrast Media and Revascularization on Organ Damage

Deterioration in renal function often affects the removal of biomarkers and their levels. The risk for significant reduction in renal function after endovascular procedures in patients with LEAD has been estimated to be around 10% and can be even higher in patients with the more advanced disease [9]. According to a study by Sigterman et al., average reduction in eGFR 1 year after endovascular intervention in symptomatic LEAD patients was 8.6 ml/min, suggesting long-term loss of kidney function [10].

One possible reason for renal injury is the effect of contrast media administration during DSA and DSA-PTA. Contrast media have been shown to directly exert a cytotoxic effect on renal tubular cells and to indirectly induce tubular hypoxia by reducing the renal blood flow and by increasing oxygen demand in the medulla [3]. Mitochondrial damage and rise in oxygen demand enhances reactive oxygen species (ROS) formation, which further damages renal tubular cells through ischemia-reperfusion injury (IRI) when oxygen supply is improved [3, 11]. However, as a significant decrease in renal function has been shown to ensue also when contrast media is not administered during angiography, other possible mechanisms reducing renal function have to be considered [12]. LEAD patients often have several concurrent comorbidities that may be exacerbated and may play a role in how organ damage manifests itself after angiographic procedure. For example, heart failure, an important risk factor of contrast media induced kidney injury, may through reduced cardiac output and venous congestion reduce perfusion of the kidneys, thus, activating renin-angiotensin-aldosterone-system (RAAS) and trigger a proinflammatory state [13]. Other such common comorbidities are diabetes, hypertension, chronic kidney disease, renal artery atherosclerosis (estimated to be present in 30-40% of LEAD patients), and acute infections that may directly or through medical therapy play a significant role [14].

Following limb revascularization IRI has been shown to occur as an immediate reaction to improved oxygenation as ROS generation is induced in skeletal muscle due to an imbalance within the antioxidant system and dysfunctional mitochondria [15]. Oxidative stress, however, induces inflammatory response that enhances leucocyte recruitment, adhesion, and activation, and is followed by a release of proinflammatory cytokines into the systemic circulation [15]. Systemic inflammatory reactions may explain elevated levels of cardiovascular complications and mortality, but also markedly increased major adverse limb events following endovascular procedures [15, 16]. This is supported by the fact that high baseline CRP values have been shown to be predictive for the risk of secondary interventions, such as open surgical procedures [17]. In our study, the patients who had received stents and completed the study had significantly higher hs-CRP levels after endovascular procedure. However, as a significant increase in hs-CRP from baseline was seen in both groups, no significant difference in hs-CRP levels was found between the groups (Table 2). Thus, RIPC did not curtail the rise in overall inflammatory response.

### 4.2. Oxidative Stress and Inflammatory Response

Oxidative stress and inflammation are well-known factors behind formation and progression of atherosclerosis, evidenced by increased synthesis of proinflammatory cytokines and oxidation of proteins and lipids in the vascular wall. Markers for oxidative stress and inflammation have been proposed as potential targets for diagnosing LEAD and evaluating its course [18, 19]. Although there was noted no statistically significant difference in oxidative stress markers between the RIPC group and the control group in our study, the decrease in the isoprostanes-creatinine ratio, a marker of lipid peroxidation, vasoconstriction, and platelet aggregation were noted only in the RIPC group, indicating the possible reduction in oxidative stress.

Myeloperoxidase (MPO), an enzyme largely produced by activated neutrophils and macrophages, is mainly considered to be a regulator of inflammatory response [20]. In our study, hs-CRP and MPO levels increased significantly in both study groups 24 hours after endovascular procedure. Intriguingly, even though there was no statistically significant difference between the groups, the increase in inflammatory markers was more pronounced in the RIPC group. Controversial at first sight, increased inflammatory response following RIPC has also been reported earlier [21, 22]. Albrecht et al. found that RIPC procedure vs. control increased MPO activity in right atrial tissue and upregulated serum cytokines in patients who had undergone cardiopulmonary bypass, while a concurrent decrease occurred in troponin T levels [21]. They suggested that even though deleterious in chronic excess, increased neutrophil numbers at an early time point in a short time frame may be not associated with negative outcome. Rather, it may positively influence the affected tissue during the initial reperfusion phase as both pro- and anti-inflammatory cytokine functions may be needed to precondition the target organ [21]. This is further supported by our finding: the levels of IL-18, a well-known proinflammatory cytokine associated with atherosclerosis, coronary artery disease, and myocardial IRI, increased only in the sham group, but not in the RIPC group [23]. This indicates that although there was increased inflammatory response after RIPC procedure, it need not be associated with increased cardiovascular risk. Rather, the contrary is likely, since proinflammatory cytokine downstream of the inflammatory pathway did not increase following RIPC procedure.

### 4.3. Adiponectin

Adiponectin, a high concentration plasma protein that is primarily produced in the adipose tissue, has been proposed to exert an anti-inflammatory and antiapoptotic effects and to increase insulin sensitivity [24, 25]. Through binding to the membrane-bound protein T-cadherin present in the vasculature, including endothelium and smooth muscle cells, adiponectin has been shown to play also a critical role in revascularization after chronic ischemia and to protect against neointimal and atherosclerotic plaque formation [26, 27]. However, contrary to findings in cellular and animal models, high levels of adiponectin have been shown to independently predict both all-cause and cardiovascular mortality in many different clinical settings, including patients with coronary artery disease, chronic kidney disease, and LEAD [28, 29]. The reasons for these controversial results are still unknown. Relative resistance to adiponectin in metabolically active organs, including the vasculature and heart, and the possible role of adiponectin as a marker for increased natriuretic peptides, due to their strong correlation with it, have been suggested in some studies [28–30]. In our study, only in the control group, but not in the RIPC group, significant rise in adiponectin levels was noted. Since we did not find a statistically significant change or even increase in NT-proBNP levels in either study group, the increase in adiponectin levels cannot be explained by the change of NT-proBNP levels. As the change of adiponectin levels was revealed in both ITT and PP analyses, but not after adjusting to baseline levels in the PP population, some of the observed difference could be ascribed to the difference in adiponectin levels already present at baseline. However, since randomisation is considered to equalize all random difference at recruitment, the difference revealed in ITT analysis can be considered as a true effect of RIPC.

Under increased inflammatory and oxidative stress conditions, adiponectin production in skeletal muscle, liver, and cardiomyocytes is upregulated [31–33]. It has been shown that it accumulates in damaged tissues as the result of leakage from the damaged endothelial barrier [34]. Massip-Salcedo et al. have previously shown that adiponectin levels increased after ischemia-reperfusion injury in steatotic rat livers compared to sham group. When ischaemic preconditioning stimulus was applied under same conditions, adiponectin levels were significantly lower [33]. In addition, with preconditioning stimulus, reduction of oxidative stress markers and reduced hepatic injury was also described. Even though serum adiponectin levels were higher in ischaemia-reperfusion group, no correlation between circulating adiponectin levels and hepatic adiponectin levels was described by the authors [33]. The reason for the observed difference in serum adiponectin levels with our study might be relatively modest preconditioning stimulus, low sample size, or the lack of involvement of skeletal muscle in inflammatory and oxidative stress condition that LEAD patients represented in our study. Whether this translates to lower cardiovascular and all-cause mortality in the RIPC vs. sham group, due to the difference in adiponectin levels, is yet to be answered in future studies.

### 4.4. The Effect of RIPC on Kidneys

Significant improvement in medullary and cortex oxygenation following the RIPC procedure has been shown in humans [35]. Previous large randomised trials of the effect of RIPC on renal biomarkers and kidney function have led to promising results [36]. However, often conflicting results have been found due to heterogeneity of study designs and populations. It has been suggested that low-risk patients and procedures might not provoke the expected difference seen in the case of high or intermediate risk procedures [37]. We found a significant increase in creatinine levels in both study groups. Although, in primary analysis, eGFR decreased only in the sham group, we cannot conclude that RIPC ameliorated the decrease of eGFR as there was no statistically significant difference between the groups in any performed analysis. As eGFR and creatinine are considered relatively delayed markers for acute changes in kidney function, we included some of the proposed earlier organ damage markers in this study.

Serum neutrophil gelatinase-associated lipocalin (NGAL) has been shown to be an early predictor of kidney injury as it is released from tubular epithelium to distal nephrons after toxic or ischaemic injury has been afflicted [38]. In addition, NGAL is also expressed by neutrophils, epithelial cells, liver, and atherosclerotic plaques [39]. Kidney injury molecule-1 (KIM-1), an indicator of renal tubular damage, is released from proximal tubules. It regulates the regeneration and repair of tubular epithelial cells after ischaemic or toxic injury and has been lately demonstrated to be protective in the early stages of kidney injury [40]. In our study, the increase in NGAL levels was observed in both study groups, but without a significant change between the RIPC group and the sham group in primary analysis. After adjusting to baseline values, a significant increase in serum NGAL levels compared to control was seen in patients who completed the study. Intriguingly, a significant increase in urinary KIM-1 occurred also in the RIPC group, but not in the sham group. However, no change in urinary L-FABP, which is exclusively present in the proximal tubule and is released in the setting of oxidative stress and ischaemia [41], was seen in our study. As only KIM-1 and NGAL, but not L-FABP levels, were elevated, changes seen in the RIPC group cannot be ascribed to markedly increased damage to proximal tubules. Rather, they serve as indicators of changes in inflammatory response, which could even be beneficial.

### 4.5. Cardiac Biomarkers

We did not find any significant difference between the sham and the RIPC groups in the cardiac markers for high-sensitive troponin T (hs-Troponin T) or N-terminal pro b-type natriuretic peptide (NT-proBNP) 24 hours after endovascular procedure. However, a significant decrease of CK-MB mass was noted in the control group in primary analysis. As CK-MB mass is considered to be a less sensitive marker for cardiac damage than hs-troponin T and is known to be elevated in muscular diseases, the change of CK-MB mass in both groups might indicate general improvement in skeletal muscle health after DSA and DSA-PTA under conditions of improved blood flow. Moreover, as the difference between the groups was revealed only in PP, but not in ITT analysis, other possible underlying conditions could have influenced these findings. This might explain why after adjusting to baseline values, there was no significant difference between the RIPC group and the sham group in CK-MB mass.

### 4.6. Limitations

Since, the period during which the patients were followed up in our study was only 24 hours, and we cannot state how prolonged follow-up would affect the difference seen in studied markers or relationships between the RIPC group and the sham group. As the aim of the study was to describe acute effects following RIPC procedure after endovascular procedure, we cannot make any conclusions beyond the causal dependence and relationships of the studied biomarkers.

## 5. Conclusion

Even though patients with LEAD undergoing DSA and DSA-PTA procedures receive less invasive intervention compared to surgery, significant changes do occur in the profile of organ damage, oxidative stress, and inflammation biomarkers. The reasons for obtaining this kind of result are yet to be explained. The heterogeneity of the human population can partly explain why clinical studies have so far failed to show the effectiveness of RIPC, as demonstrated in animal models. Nonetheless, RIPC as a method to modify the response to IRI stimuli and inflammation in patients with LEAD deserves further investigation.

## Figures and Tables

**Table 1:** Baseline characteristics of the population intention-to-treat analysis.

Characteristics	RIPC (*n* = 54)		SHAM (*n* = 57)		*p* value
	Mean/median	SD/IQR	Mean/median	SD/IQR	
Demographic					
Male (*n*)	39 (72.2%)		48 (84.2%)		0.193
Mean age (y)	65.5	±10.1	65.3	±11.9	0.903
Weight (kg)	76.6	±17.5	78.2	±17.1	0.620
Body mass index (kg/m^2^)	25.4	(23.0-30.0)	25.7	(23.5-29.4)	0.788
Renal function at inclusion					
eGFR < 90 (*n*) ^#^	30 (55.6%)		32 (56.1%)		1
60-89 (*n*) ^#^	20 (37.0%)		20 (35.1%)		
30-59 (*n*) ^#^	10 (18.5%)		12 (17.5%)		
History of smoking (*n*) ^†^	42 (77.8%)		42 (73.7%)		0.779
Concomitant diseases					
Stage of LEAD III or more ^‡^	27 (50.0%)		27 (47.4%)		0.930
Stage of LEAD III (*n*) ^‡^	11 (20.4%)		10 (17.5%)		
Stage of LEAD IV (*n*) ^‡^	16 (29.6%)		17 (29.8%)		
Diabetes (*n*)	12 (22.2%)		15 (26.3%)		0.779
Hypertension (*n*) ^◊^	35 (64.8%)		31 (54.4%)		0.355
Medications					
ACE inhibitors (*n*)	20 (37.0%)		16 (28.1%)		0.313
ARBs (*n*)	14 (25.9%)		11 (19.3%)		0.403
Calcium channel blockers (*n*)	18 (33.3%)		17 (29.8%)		0.691
Beta blockers (*n*)	13 (24.1%)		15 (26.3%)		0.786
Diuretics (*n*)	18 (33.3%)		14 (24.6%)		0.308
Antiagregants (*n*)	29 (53.7%)		26 (45.6%)		0.394
Anticoagulants (*n*)	1 (1.9%)		2 (3.5%)		0.591
Naftidrofuryl/pentoxifylline (*n*)	37 (68.5%)		36 (63.2%)		0.552
Statins (*n*)	20 (37.0%)		16 (28.1%)		0.381
Insulin therapy (*n*)	7 (13.0%)		8 (14.0%)		0.869
Oral antidiabetic agents (*n*)	4 (7.4%)		7 (12.3%)		0.390
PSBP (mmHg)	144.3	±21.9	139.9	±18.3	0.253
PDBP (mmHg)	78.0	±11.8	75.9	±9.8	0.324
Heart rate (bpm)	66.1	±10.2	67.6	±10.3	0.459
WBC (10^9^/L)	7.04	±2.00	6.99	±1.79	0.895
RBC (10^12^/L)	4.57	±0.44	4.60	±0.44	0.752
HGB (g/L)	136.1	±16.8	141.6	±14.4	0.067
Hct (%)	40.3	±6.5	42.0	±3.9	0.103
PLT (10^9^/L)	252.5	±85.3	231.5	±56.2	0.131
High-sensitivity troponin T (ng/L)	9.9	(6.8-15.7)	11.2	(6.6-17.6)	0.669
Creatine kinase MB mass (*μ*g/L)	1.8	(1.5-2.6)	1.9	(1.6-2.8)	0.392
N-terminal proBNP (pg/mL)	168	(93-448)	94	(50-376)	0.131
hs-CRP (mg/L)	2.54	(1.65-6.15)	3.02	(1.51-5.26)	0.841
Glucose (mmol/L)	5.1	(4.8-5.7)	5.3	(4.9-6.2)	0.233
Creatinine (*μ*mol/L)	76	(65-87)	78	(67-92)	0.899
Urea (mmol/L)	5.0	(4.3-6.6)	5.6	(4.4-6.6)	0.543
Cystatine C (mg/L)	1.11	(0.96-1.36)	1.10	(0.93-1.31)	0.807
Cholesterole (mmol/L)	4.74	±1.38	4.83	±1.39	0.733
HDL (mmol/L)	1.19	(0.97-1.56)	1.10	(0.92-1.43)	0.244
LDL (mmol/L)	2.76	(2.10-3.67)	3.01	(2.07-3.90)	0.669
TG (mmol/L)	1.33	(1.05-1.96)	1.43	(1.12-1.98)	0.452
B-2-microglobuline (*μ*g/L)	2470	(2042-2870)	2180	(1870-2780)	0.137
eGFR (mL/min/1.73 m^2^)	86	(71-95)	91	(68-100)	0.392
Adiponectine (ng/mL)	5808	(3419-8507)	5619	(3327-7654)	0.660
Myeloperoxidase (ng/mL)	58.7	(33.5-85.3)	52.5	(30.7-88.8)	0.543
NGAL (ng/mL)	81.8	(63.5-101.5)	71.9	(65.0-83.2)	0.080
Oxidized low-density lipoprotein (U/L)	56.0	(45.7-73.7)	65.5	(44.0-79.3)	0.291
Kidney injury molecule 1 (pg/mL)	1406	(738-2354)	1440	(839-2407)	0.927
L-FABP (ng/mL)	0.85	(0.67.1.52)	0.87	(0.62-1.51)	0.863
Isoprostane/creatinine ratio (ng/mmol)	41.0	(33.1-50.1)	45.5	(32.9-61.0)	0.318
IL-18 (pg/mL)	276	(231-361)	283	(201-348)	0.864

†: current and ex-smokers; ‡: stage of LEAD by Fontaine's classification; ◊: on medication; #: ml/min/1.73 m^2^; y: years of age; LEAD: lower extremity arterial disease; eGFR: estimated glomerular filtration rate; ACE: angiotensin-converting enzyme; ARB: angiotensin receptor blocker; PSBP: peripheral systolic blood pressure; PDBP: peripheral diastolic blood pressure; L-FABP: liver-type fatty acid-binding protein; NGAL: neutrophil gelatinase-associated lipocalin; hs-CRP: high-sensitivity C-reactive protein.

**Table 2:** Mean change of organ damage biomarkers in the groups of the per-protocol population with respect to stenting.

	RIPC			SHAM			*p* value^+^
	*p* value	Stent		*p* value	Stent		
		No	Yes		No	Yes	
		Mean	Mean		Mean	Mean	
*Δ*hs-TropT (ng/L)	0.51	-0.09	-0.15	0.48	-0.31	-0.54	0.56
*Δ*CK-MBm (*μ*g/L)	0.65	-0.45	-0.08	0.26	-0.36	-0.56	0.33
*Δ*NT-proBNP (pg/mL)	0.66	-54.0	-39.6	0.34	-51.0	-23.0	0.87
*Δ*hs-CRP (mg/L)	0.036	0.97	7.56	<0.0001	-0.93	2.57	0.059
*Δ*Glucose (mmol/L)	0.76	0.25	0.38	0.88	-0.09	0.31	0.62
*Δ*Creatinin (*μ*mol/L)	0.46	1.46	3.95	0.47	3.33	1.37	0.80
*Δ*Urea (mmol/L)	0.59	0.07	0.31	0.039	0.35	-0.07	0.76
*Δ*Cystatin-C (mg/L)	0.20	0.01	0.03	0.49	0.00	-0.02	0.19
*Δ*Beta-2 microglobuline (*μ*g/L)	0.20	14.2	142.3	0.69	-13.8	-3.7	0.10
*Δ*eGFR (mL/min/1.73m^2^)	0.47	-0.8	-3.1	0.37	-2.9	-1.2	0.95
*Δ*Adiponectin (ng/mL)	1	268	-221.2	0.44	204	46	0.72
*Δ*IL-18 (pg/mL)	0.24	0.7	11.6	0.88	7.5	5.0	1
*Δ*MPO (ng/mL)	0.53	23.3	39.9	0.075	0.5	27	0.087
*Δ*NGAL (ng/mL)	0.90	9.9	11.6	0.38	5.4	5.1	0.089
*Δ*ox-LDL (U/L)	0.89	-1.8	-0.4	0.47	-1.0	0.8	0.67
*Δ*KIM-1 (pg/mL)	0.56	1569	1741	0.44	881	645	0.17
*Δ*L-FABP (ng/mL)	0.72	-0.06	-0.04	0.084	0.11	-0.14	0.81
*Δ*Isoprostanes/creatinine (ng/mmol)	0.026	-7.8	3.5	0.005	-11.6	2.1	

*Δ*: mean change; ^+^: adjusted to stenting; hs-TropT: high-sensitivity troponin T; CK-MBm: creatine kinase MB mass; NT-proBNP: N-terminal proBNP; Glc: glucose; MPO: myeloperoxidase; eGFR: estimated glomerular filtration rate; NGAL: neutrophil gelatinase-associated lipocalin; oxLDL: oxidized low-density lipoprotein; hs-CRP: high-sensitivity C-reactive protein; KIM-1: kidney injury molecule 1; L-FABP: liver-type fatty acid-binding protein.

## Data Availability

Data available on request through the corresponding author (karl.kuusik@kliinikum.ee).

## Supplementary Materials

Supplementary Materials Table S1: baseline characteristics of the population of per-protocol analysis.
