# Supplementary material for: The Role of RIPC in Preventing Organ Damage, Inflammation, and Oxidative Stress during Lower Limb DSA: A Randomised Controlled Trial
Source: Oxid Med Cell Longev. 2021 Dec 8;2021:6043550. doi: 10.1155/2021/6043550 (PMC8674049; doi:10.1155/2021/6043550)
Supplement: Supplementary Materials — Table S1: baseline characteristics of the population of per-protocol analysis. [file 6043550.f1.docx]

**Table S1.** Baseline characteristics of the population of per-protocol analysis.

|  | RIPC (n=46) | | SHAM (n=54) | | p-value |
| --- | --- | --- | --- | --- | --- |
| Characteristics | Mean/ median | SD/IQR | Mean/ median | SD/IQR |  |
| Demographic |  | | | | |
| Male (n) | 33 (71.7%) | | 47 (87.0%) | | 0.098 |
| Mean age (y) | 66.1 | ±10.3 | 65.0 | ±11.4 | 0.605 |
| Weight (kg) | 75.2 | ±17.3 | 78.0 | ±16.7 | 0.421 |
| Body mass index (kg/m^2^) | 25.4 | (22.7-30.0) | 25.3 | (23.5-29.4) | 0.661 |
| Renal function at inclusion |  | |  | |  |
| eGFR <90 (n) ^#^ | 27 (58.7%) | | 30 (55.6%) | | 0.910 |
| 60-89 (n) ^#^ | 19 (41.3%) | | 20 (37.0%) | |  |
| 30-59 (n) ^#^ | 8 (18.5%) | | 10 (18.5%) | |  |
| History of smoking (n) ^†^ | 35 (76.1%) | | 41 (75.9%) | | 1 |
| Concomitant diseases |  | |  | |  |
| Stage of LEAD III or more ^‡^ | 23 (50.0%) | | 25 (46.3%) | | 0.866 |
| Stage of LEAD III (n) ^‡^ | 9 (19.6%) | | 10 (18.5%) | |  |
| Stage of LEAD IV (n) ^‡^ | 14 (30.4%) | | 15 (27.8%) | |  |
| Diabetes (n) | 10 (21.7%) | | 13 (24.1%) | | 0.970 |
| Hypertension (n) ^◊^ | 32 (69.6%) | | 28 (51.9%) | | 0.110 |
| Medications |  | |  | |  |
| ACE inhibitors (n) | 18 (39.1%) | | 14 (25.9%) | | 0.158 |
| ARBs (n) | 12 (26.1%) | | 11 (20.4%) | | 0.498 |
| Calcium channel blockers (n) | 18 (39.1%) | | 15 (29.8%) | | 0.229 |
| Beta blockers (n) | 12 (26.1%) | | 12 (22.2%) | | 0.652 |
| Diuretics (n) | 16 (34.8%) | | 12 (22.2%) | | 0.163 |
| Antiagregants (n) | 24 (52.2%) | | 26 (48.1%) | | 0.688 |
| Anticoagulants (n) | 1 (2.2%) | | 1 (1.9%) | | 0.909 |
| Naftidrofuryl/pentoxifylline (n) | 33 (71.7%) | | 35 (64.8%) | | 0.459 |
| Statins (n) | 18 (39.1%) | | 16 (29.6%) | | 0.385 |
| Insulin therapy (n) | 6 (13.0%) | | 8 (14.8%) | | 0.799 |
| Oral antidiabetic agents (n) | 3 (6.5%) | | 5 (9.3%) | | 0.615 |
| PSBP (mmHg) | 144.2 | ±22.2 | 138.5 | ±17.2 | 0.165 |
| PDBP (mmHg) | 77.3 | ±12.1 | 75.7 | ±9.8 | 0.481 |
| Heart rate (bpm) | 65.2 | ±9.9 | 67.0 | ±10.2 | 0.366 |
| WBC (10^9^/L) | 6.80 | ±1.74 | 6.99 | ±1.79 | 0.692 |
| RBC (10^12^/L) | 4.56 | ±0.42 | 4.58 | ±0.44 | 0.920 |
| HGB (g/L) | 135.9 | ±16.3 | 141.6 | ±14.7 | 0.075 |
| Hct (%) | 41.0 | ±4.3 | 42.0 | ±4.0 | 0.210 |
| PLT (10^9^/L) | 232.5 | (187.8-280.2) | 219.5 | (190.2-264.2) | 0.491 |
| High-sensitivity troponin T (ng/L) | 9.9 | (6.9-16.8) | 11.1 | (6.5-16.8) | 1.0 |
| Creatine Kinase MB Mass (µg/L) | 1.8 | (1.5-2.8) | 2.0 | (1.6-3.1) | 0.319 |
| N-terminal proBNP (pg/mL) | 200 | (92-463) | 89 | (45-292) | 0.037 |
| hs-CRP (mg/L) | 2.23 | (1.52-5.24) | 2.81 | (1.51-4.91) | 0.567 |
| Glucose (mmol/L) | 5.1 | (4.8-5.7) | 5.3 | (4.8-6.2) | 0.508 |
| Creatinine (μmol/L) | 78 | (65-92) | 77 | (67-92) | 0.796 |
| Urea (mmol/L) | 5.0 | (4.4-6.6) | 5.5 | (4.4-6.6) | 0.759 |
| Cystatine C (mg/L) | 1.11 | (0.97-1.36) | 1.09 | (0.93-1.26) | 0.446 |
| Cholesterole (mmol/L) | 4.66 | ±1.38 | 4.85 | ±1.42 | 0.517 |
| HDL (mmol/L) | 1.17 | (0.96-1.55) | 1.12 | (0.94-1.45) | 0.583 |
| LDL (mmol/L) | 2.70 | (2.07-3.63) | 3.02 | (2.05-3.91) | 0.504 |
| TG (mmol/L) | 1.30 | (0.98-2.06) | 1.43 | (1.1-1.98) | 0.358 |
| B-2-microglobuline (μg/L) | 2470 | (2080-2840) | 2145 | (1830-2750) | 0.058 |
| eGFR (mL/min/1.73m^2^) | 84 | (68-94) | 91 | (69-100) | 0.174 |
| Adiponectine (ng/mL) | 6322 | (3769-8523) | 5541 | (3327-9406) | 0.484 |
| Myeloperoxidase (ng/mL) | 57.8 | (32.4-81.8) | 51.4 | (30.2-81.6) | 0.575 |
| NGAL (ng/mL) | 82.1 | (65.5-103.0) | 71.8 | (61.12-80.7) | 0.019 |
| Oxidized low-density lipoprotein (U/L) | 55.6 | (45.3-71.1) | 65.1 | (43.5-79.3) | 0.212 |
| Kidney injury molecule 1 (pg/mL) | 1392 | (733-2215) | 1455 | (870-2432) | 0.416 |
| L-FABP (ng/mL) | 0.85 | (0.61-1.43) | 0.81 | (0.62-1.42) | 0.736 |
| Isoprostane/creatinine ratio (ng/mmol) | 40.8 | (30.2-50.5) | 44.6 | (32.9-61.0) | 0.287 |
| IL-18 (pg/mL) | 269 | (230-364) | 280 | (196-334) | 0.833 |

†- current and ex-smokers; ‡- Stage of LEAD by Fontaine´s classification; ^◊^- on medication; ^#^- ml/min/1.73m^2^; y – years of age; PAD- peripheral arterial disease; eGFR- estimated glomerular filtration rate; ACE- angiotensin-converting enzyme; ARB- angiotensin receptor blocker; PSBP- peripheral systolic blood pressure; PDBP- peripheral diastolic blood pressure; L-FABP: liver-type fatty acid binding protein; NGAL: neutrophil gelatinase-associated lipocalin; hs-CRP: high-sensitivity C-reactive protein
